# Young patients’ involvement in a composite endpoint method development on acceptability for paediatric oral dosage forms

**DOI:** 10.1186/s40900-023-00520-8

**Published:** 2023-11-29

**Authors:** Sibylle Reidemeister, Begonya Nafria Escalera, Daniel Marín, Jan Balayla, Ingrid Klingmann, Viviane Klingmann

**Affiliations:** 1 Global Drug Development, Novartis Pharma AG, WSJ-188 10 001, 4056 Basel, Switzerland; 2 KIDS Barcelona, Institut de Recerca Sant Joan de Déu, Santa Rosa 39-56, 08950 Esplugues de Llobregat, Spain; 3 Patient Engagement in Research Coordinator, Institut de Recerca Sant Joan de Déu, Santa Rosa 39-56, 08950 Esplugues de Llobregat, Spain; 4 Innovation Department, Hospital Sant Joan de Déu de Barcelona, Passeig de Sant Joan de Déu 2, 08950 Esplugues de Llobregat, Spain; 5 Pharmaplex Bv, Avenue Saint-Hubert 51, 1970 Wezembeek-Oppem, Belgium; 6 Department of General Pediatrics, Neonatology and Pediatric Cardiology, University Children’s Hospital Düsseldorf, Medical Faculty, Heinrich-Heine-University, Moorenstrasse 5, 40225 Düsseldorf, Germany

**Keywords:** Acceptability, Composite endpoint on acceptability, Method development, Paediatric drug development, Palatability, Patient involvement, Swallowability, YPAG

## Abstract

**Background:**

In line with the European Paediatric Regulation, the European Medicines Agency (EMA) asks for investigation of a medicine’s acceptability in paediatric medicines development. A standardised acceptability testing method combining the outcome of “swallowability” and “palatability” assessments to a “composite endpoint on acceptability” was recently developed. Before this method’s suitability for selection of the most acceptable drug formulation of a new medicine for children can be broadly recommended, the acceptance and relevance of such established acceptability needs the critical review and input from young patients with understanding of the medicines development methodology. The benefit of involving patients in drug product development, clinical research and innovation is well established.

**Methods:**

During a focus group meeting with the KIDS Barcelona (young people advisory group, age 16–23 years) the suitability of the “composite endpoint on acceptability” methodology was assessed. Via electronic questionnaires the importance of involving patients in the medicines development and in the acceptability method development was investigated. Questions on how best to determine palatability and swallowability were asked. The relevance of all EMA-listed acceptability elements was assessed via coloured and numbered stickers and questionnaires.

**Results:**

The results showed that the involvement of young people in the medicines and acceptability method development was rated high. The group worked out that a 5-point smiley Likert Scale is preferred for assessing acceptability by 6–11 year old patients, while a Visual Analogue Scale is preferred for collecting adolescents’ opinion. The ranking of the EMA-listed acceptability elements showed that palatability and swallowability are the most relevant parameters, while colour of the medicine was rated as least relevant. These results, established face-to-face, were confirmed in a repeat of the ranking through an electronic questionnaire, completed by the participants individually and remotely, 5 weeks later.

**Conclusion:**

This work reinforced the need and value to involve young people in the medicines lifecycle, and specifically in this acceptability method development. As next step other focus group meetings with more young people from different European countries are planned.

**Supplementary Information:**

The online version contains supplementary material available at 10.1186/s40900-023-00520-8.

## Abstract (plain language summary)

Before a new medicine is authorized, its acceptability by children must be investigated according to law. An acceptability testing method combining the outcomes of “swallowability” and “palatability” assessments was recently developed. During a focus group meeting with KIDS Barcelona (young people advisory group, age 16–23 years) their opinion on the suitability of the method and the relevance of patient engagement in the medicines development process were assessed with paper-based and electronic questionnaires. Questions on how best to determine palatability and swallowability were asked. The importance of different elements that typically affect acceptability was rated. The order of relevance of those listed acceptability elements was assessed using coloured and numbered stickers and questionnaires. The results showed that the involvement of young people in the medicines and acceptability method development was rated high. The group worked out that a 5-point smiley Likert Scale that allows for marking a choice between total agreement and total disagreement is preferred for assessing acceptability by 6–11 year old patients. A Visual Analogue Scale (scale consisting of a 10 cm long line on which a mark has to be placed at the desired position, between total agreement and total rejection) is preferred for collecting adolescents’ (12–18 years) opinion. The ranking of acceptability elements showed that palatability and swallowability of a new medicine are the most relevant parameters, and colour the least. The clarity of the outcome reinforced the benefit of involving young people in the development of medicines relevant for children.

**Supplementary Information:**

The online version contains supplementary material available at 10.1186/s40900-023-00520-8.

## Introduction/background

Evaluation of patient acceptability of a paediatric medicinal product is an integral part of the pharmaceutical and clinical development of a medicine for children as requested in EU [1], US [2], an ICH [3] Guidance. Patient acceptability of a medicinal product should preferably be demonstrated in children themselves as part of a clinical trial design involving the proposed medicinal product.

The Paediatric Regulation EU 1901/2006 [4] requests that the drug developer prepares a Paediatric Investigation Plan that specifies the timing and measures proposed to assess quality, efficacy and safety of the new medicinal product in all subsets of the paediatric population where appropriate. In addition, the Paediatric Investigation Plan is supposed to contain any measures to adapt the formulation of the medicinal product for different subsets of the paediatric population, from 0 to younger than 18. The European Medicines Agency (EMA) “Guideline on pharmaceutical development of medicines for paediatric use” [1] states: “Patient acceptability is likely to have a significant impact on patient adherence and consequently, on the safety and efficacy of a medicinal product. Acceptability is determined by the characteristics of the product and the user. The product aspects relate to pharmaceutical characteristics such as palatability, swallowability (e.g. size, shape, texture) …” [1].

“Different acceptability methods have been described in literature, which resulted in different outcomes when testing the same medicine in the same patient population” [1]. As knowledge on acceptability testing methodology is still fragmented and an internationally accepted standard method has not yet been developed, the choice of the acceptability method and the acceptance criteria are left to the applicant [1].

Given the importance of the need, an increase of newly developed acceptability assessment methods and evaluation criteria can be observed [5, 6].

**Fig. 1:**
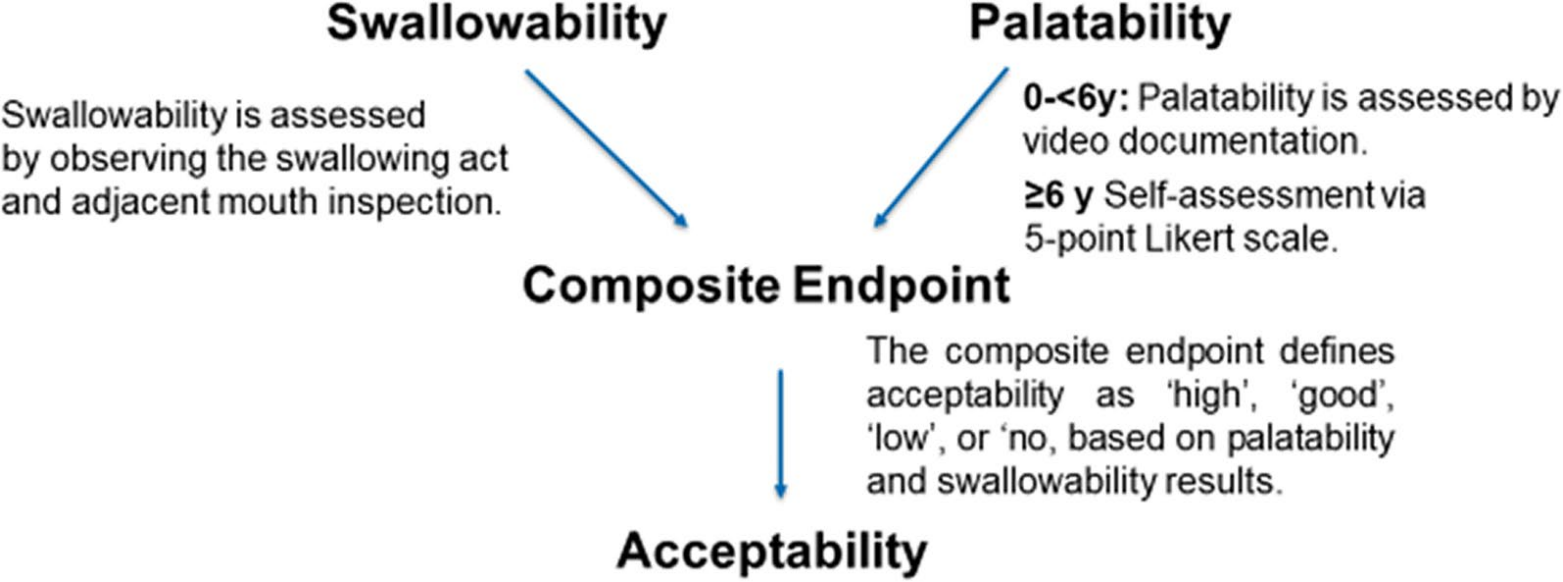
Composite endpoint on acceptability

In 2021, a public–private multi-stakeholder research group (paediatrician, statistician, pharmacist, and clinical researcher) developed a new acceptability method by statistically combining the outcome of “swallowability” and “palatability” assessments to a new so-called “composite endpoint on acceptability” (Fig. 1) for oral dosage form development [7].

According to the underlying validated method [8–14] swallowability is assessed by observing the act of swallowing and a rapid mouth inspection by a trained investigator, according to a given scoring scheme. Palatability can be described as a physical expression, gestures and—in older children—also as verbal expression—in response to the appearance, smell, taste, after taste and mouth feel (e.g., texture, cooling, heating, trigeminal response) of an oral medicine [5, 15]. Assuming that a combination of swallowability and palatability would describe acceptability more precisely, the composite endpoint on acceptability was developed defining acceptability as ‘high’, ‘good’, ‘low’, or ‘no’ based on swallowability and palatability results.

Although the “composite endpoint on acceptability” method has been developed by physicians and clinical researchers, the most relevant stakeholder group to assess the suitability of this tool are the patients. The potential benefit that patients can add with their advice on drug product development, clinical research and innovation is fundamental to ensure that the scales and methods that are developed are appropriate, understandable, and suited. Consequently, the activities to involve patients are growing, however, most of the published work focuses on clinical development and on adults.

In this project the adolescents and young adults aged 16–23 years that have contributed belong to the KIDS Barcelona Young Person’s Advisory Group (YPAG, for details please see Additional file 1: GRIPP 2 Short form). YPAGs typically are formed of 10–20 members aged between 8 and 21 years and have experience of taking part in a clinical trial and/or have a chronic condition or have a general interest in health, research, and science [16]. The KIDS Barcelona group (which comprises 20 members in total) was established in 2015 by Sant Joan de Déu Research Foundation after having offered to its members a training in health diagnosis, clinical research, clinical trials, and innovation. The team is regularly involved in different kind of activities: review and validation of assent forms, co-creation of informative and educational resources (booklets, videos, etc.), co-design of awareness campaigns in the field of paediatric clinical research, co-creation in the field of innovation projects, etc.

In 2017, the European Young Person’s Advisory Groups Network (eYPAGnet, www.eypagnet.eu) was established with the aim to facilitate the involvement of young people in clinical research based on the value that European countries’ diversity brings to research projects but also ensuring the contribution in consultation activities of young people experts. This network is recognized by Enpr-EMA (European Network of Paediatric Research of the European Medicines Agency). YPAGs were created based on the principle that children should transform from research subjects into research partners.

The goal of this publication is to describe how adolescents and young adults could be involved in scientific method development, namely the suitability of the “composite endpoint on acceptability” method. After this first experience with the KIDS Barcelona group, the activity can be further elaborated upon to be rolled out to a larger group of YPAGs. The paediatric formulation research group and 11 members of the KIDS Barcelona YPAG, collaborated to involve patients in the suitability assessment of this new acceptability method (“composite endpoint on acceptability”) and to assess in a systematic way whether the methodologies are meeting the patients’ needs and expectations.

## Methods

During a focus group meeting of the research group and the KIDS Barcelona YPAG in December 2022 in Barcelona, Spain, first the suitability of the clinician-observed and patient self-assessed “composite endpoint on acceptability” methodology was reviewed and assessed by 11 (6 female and 4 male) adolescents (n = 5) and young adults (n = 7) (16–23; mean age: 20.63y; for sociodemographic details please see Table 1); six of them are patients. Terms like acceptability, swallowability and palatability of an oral drug formulation were explained. Subsequently, the new approach to combine the results from the assessments of swallowability and palatability as acceptability by the newly developed “composite endpoint on acceptability” method was described.

**Table 1:** Sociodemographic details of the involved adolescents and young adults of Barcelona KIDS YPAG

Age (years)	Gender	Are you a patient?
18	Female	No
16	Female	No
17	Male	No
19	Female	Yes
20	Male	Yes
17	Female	Yes
17	Female	Yes
21	Male	No
21	Female	Yes
23	Male	Yes
17	Male	No

Young people were asked to rate the importance of paediatric patient involvement presented in the following 2 questions on a 10 (highest) to 1 (lowest) scale in a bilingual (English and Spanish) electronic questionnaire (Google Form):

1. How do you rate the importance of involving young people in the medicines development process in general? (Cómo valoras de importante la involucración activa de los jóvenes en el proceso de investigación de un medicamento?)
2. How do you rate the importance of involving young people in the acceptability method development for paediatric galenic formulations? (Cómo valoras de importante la involucración activa de los jóvenes en el proceso de diseño de un método para medir la aceptabilidad de una formulación de un medicamento?)

The next question related to the age group that should be involved in acceptability method development and in self-assessment of acceptability. The young people were asked to answer the following two questions by selecting an age group in the electronic questionnaire:

1. Which age group should give input into acceptability method development? (De los siguientes grupos de edad, cuál debería participar en el desarrollo/diseño de un método de estudio de la aceptabilidad de la formulación de un medicamento?)
  12–18 years (12–18 años)
  10–18 years (10–18 años)
  8–18 years (8–18 años)
  6–18 years (6–18 años)
2. Which age group of young people should do self-assessment of the acceptability of galenic formulations? (De los siguientes grupos de edad, cuál debería participar en la autoevaluación de un método de estudio de la aceptabilidad de la formulación de un medicamento?)
  12–18 years (12–18 años)
  10–18 years (10–18 años)
  8–18 years (8–18 años)
  6–18 years (6–18 años)

Subsequently, the participants were asked to give their opinion on six questions concerning palatability on a 10 (highest) to 1 (lowest) rating scale in an electronic questionnaire:

1. How suitable do you consider the 5-point smiley Likert Scale to assess palatability in the age group 12–18 years? (A qué nivel consideras adecuada una escala Likert de 5 puntos para evaluar la adaptabilidad en el grupo de edad de 12 a 18 años?)
2. How suitable do you consider the 5-point smiley Likert Scale to assess palatability in the age group 6–11 years? (A qué nivel consideras adecuada una escala Likert de 5 puntos para evaluar la adaptabilidad en el grupo de edad de 6 a 11 años?)
3. In your opinion, how attractive is the Visual Analogue Scale of 10 cm for the assessment of palatability for the age group 12–18 years? (En tu opinión, valora la Escala Visual Analógica de 10 cm para la evaluación de la palatabilidad como atractiva para el grupo de edad de 12 a 18 años?)
4. In your opinion, how attractive is the 5-point Likert Scale without any icons for the assessment of palatability for the age group 12–18 years? (En tu opinión, valora la Escala Likert de 5 puntos sin íconos para la evaluación de la palatabilidad como atractiva para el grupo de edad de 12 a 18 años?)
5. In your opinion, how attractive is the 5-point Likert Scale with other icons for the assessment of palatability for the age group 12–18 years? (En tu opinión, valora la Escala Likert de 5 puntos con otros iconos para la evaluación de la palatabilidad como atractiva para el grupo de edad de 12 a 18 años?)
6. In your opinion, how attractive is the YES–NO question for the assessment of palatability for the age group 12–18 years? (En tu opinión, valora la Pregunta SI-NO para la evaluación de la palatabilidad como atractiva para el grupo de edad de 12 a 18 años?)

This was followed by the request to answer 6 questions on swallowability on the 10 (highest) to 1 (lowest) scale in the electronic questionnaire:

7. In your opinion, how suitable is the investigator-assessed swallowability method for young people aged 12–18 years? (En tu opinión, valora si el método de deglución evaluado por el investigador es adecuado para jóvenes de 12 a 18 años?)
8. In your opinion, how suitable is the investigator-assessed swallowability method for young people from 6 to 11 years? (En tu opinión, valora si el método de deglución evaluado por el investigador para jóvenes de 6 a 11 años es adecuado?)
9. In your opinion, rate the Visual Analogue Scale of 10 cm for the assessment of swallowability for young people: (En tu opinión, valora la Escala Visual Analógica de 10 cm para la evaluación de la deglución como atractiva para los jóvenes:)
10. In your opinion, rate the 5-point Likert Scale without any icons for the assessment of swallowability for young people: (En tu opinión, valora la Escala de Likert sin iconos para la evaluación de la deglución como atractiva para los jóvenes:)
11. In your opinion, rate the 5-point Likert Scale using other icons for the assessment of swallowability for young people: (En tu opinión, valora la Escala de Likert con otros iconos para la evaluación de la deglución como atractiva para los jóvenes:)
12. In your opinion, rate the YES–NO question for the assessment of swallowability for young people: (En tu opinión, valora la pregunta SI-NO para la evaluación de la deglución como atractiva para los jóvenes:)

Then the group of young people was asked to answer the following question in the electronic questionnaire:

“Do you think that there are cultural differences to be expected when children and teenagers from different countries will answer the previous questions?” (Crees que se habrá diferencias culturales cuando los niños y adolescentes de diferentes países respondan las preguntas anteriores?) by choosing from the options:

- Yes, I expect big differences in teenagers (Sí, espero grandes diferencias en los adolescentes)
- Yes, I expect big differences in children of 6–11 years (Sí, espero grandes diferencias en niños de 6 a 11 años)
- Yes, but I expect only minor differences in teenagers (Sí, pero solo espero pequeñas diferencias en los adolescentes)
- Yes, but I expect only minor differences in children of 6–11 years (Sí, pero espero solo diferencias menores en niños de 6 a 11 años)
- No, I expect no differences in teenagers (No, no espero diferencias en los adolescentes)
- No, I expect no differences in children of 6–11 years (No, no espero diferencias en niños de 6 a 11 años)

In the second part of the meeting the patient’s view on the relevance of all eight EMA-listed elements of acceptability was assessed. The EMA parameters [1] for acceptability were presented to the young people with pictures of practical examples:

1. Palatability, swallowability (e.g., size, shape, texture)
2. Appearance (e.g., colour, shape, embossing)
3. Complexity of the modification to be conducted by the child or its caregivers prior to administration
4. The required dose (e.g., the dosing volume, number of tablets, etc.)
5. The required dosing frequency and duration of treatment
6. The selected administration device
7. The container closure system
8. The actual mode of administration to the child and any related pain or discomfort

The adolescents and young adults were asked to answer the question: “Which EMA parameter is the most important and which is the least important?” They were asked to put a green sticker for the most and a red sticker for the least relevant parameter behind the respective item presented on a flip chart. This exercise was done in person on December 16th, 2022, during the focus group meeting, and repeated individually on January 27th, 2023, to investigate the consistency of the judgements of the young people directly after the explanations within the group meeting and with a time lag of 5 weeks. The second assessment in January 2023 was done individually by the young people completing a paper questionnaire at home.

Afterwards, the adolescents and young adults were asked to rank the eight EMA guideline parameters from 1 (most) to 8 (least) important by sticking the numbers (1) to (8) behind the parameters; this was done on December 16th, 2022. On January 27th, 2023, this ranking exercise was repeated by requesting the participants to individually complete the online version of the questionnaire.

## Results

### Importance of involving patients in the medicines and acceptability method development

The importance of involving adolescents and young adults in the medicines development process and more specifically in the acceptability method development are given in Table 2. The group rated both questions as equally important, both had very high ratings with an average of 9.8 points out of 10.

**Table 2:** Importance of involving young people in the medicine’s development process and in the acceptability method development

How do you rate the importance of involving young people in the medicines development process in general?	How do you rate the importance of involving young people in the acceptability method development for paediatric galenic formulations?
10	10
10	10
10	10
10	10
10	10
10	10
10	10
10	10
10	10
10	9
8	9
9.82	9.82

### Age group to be involved in acceptability method development and in self-assessment of acceptability

**Fig. 2:**
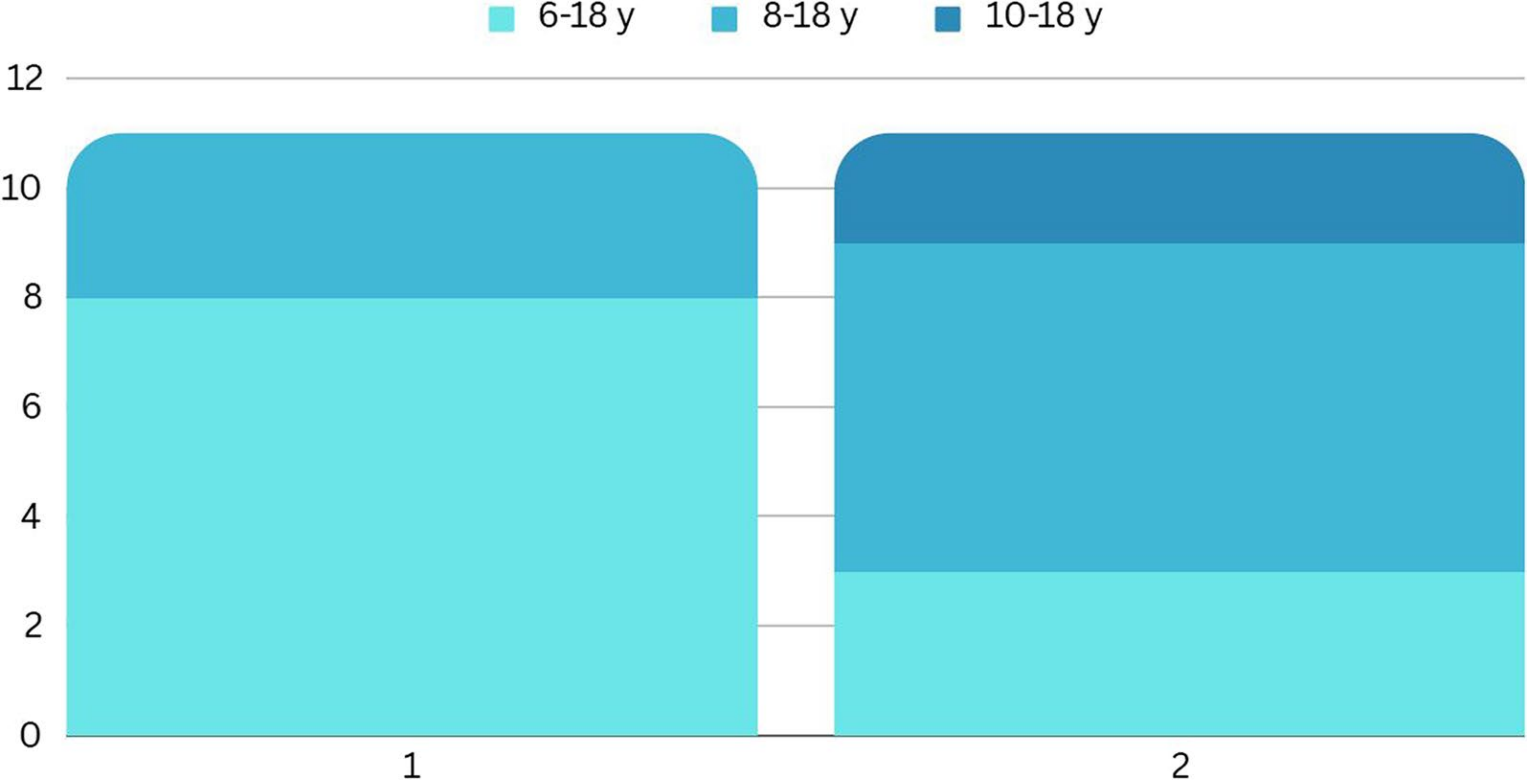
Age group to be involved in acceptability method development and in self-assessment of acceptability. Question 1: From which age group input into acceptability method development should be enabled? Question 2: Which age group of young people should do self-assessment of the acceptability of galenic formulations?

The results on the question which age group should be involved in acceptability method development and in self-assessment of acceptability testing are given in Fig. 2.

Most young people suggested that 6–18-year-old patients should give input into acceptability method development and that self-assessment of the acceptability of galenic formulations is best suited for the age group 8–18 years.

### How to measure palatability

The results of the six questions on how to measure palatability are given in Table 3. The young people rated on using a 5-point Likert Scale with or without icons and for different age-groups, or a visual analogue scale or just a simple YES–NO.

**Table 3:** Questions on how to measure palatability (10 (highest) to 1 (lowest) rating scale)

How suitable do you consider the 5-point smiley Likert Scale to assess palatability in the age group 12–18 years?	How suitable do you consider the 5-point smiley Likert Scale to assess palatability in the age group 6–11 years?	In your opinion, how attractive is the Visual Analogue Scale of 10 cm for the assessment of palatability for the age group 12–18 years?	In your opinion, how attractive is the 5-point Likert Scale without any icons for the assessment of palatability for the age group 12–18 years?	In your opinion, rate the 5-point Likert Scale with other icons for the assessment of palatability as attractive for the age group 12–18 years?	In your opinion, rate the YES–NO question for the assessment of palatability as attractive for the age group 12–18 years?
8	6	1	3	8	10
7	9	8	8	8	9
8	6	8	8	7	9
1	4	10	4	4	7
5	10	9	7	6	1
3	8	10	4	3	4
6	10	8	9	9	6
2	10	9	7	7	3
6	10	7	5	10	5
5	9	9	9	9	7
5	8	9	8	7	8
5.09	8.18	8.00	6.55	7.09	6.27

The results demonstrate a high value for the suitability of the 5 point smiley Likert Scale and seem to suggest a preference for a Visual Analog Scale of 10 cm for young people.

### How to measure swallowability

The results of the six questions on how to measure swallowability are given in Table 4. The young people were asked for their rating on using the investigator-assessed swallowability method for different age-groups, a 5-point Likert Scale with or without icons and for different age-groups, or a Visual Analogue Scale or just a simple YES–NO.

**Table 4:** Questions on how to measure swallowability (10 (highest) to 1 (lowest) rating scale)

In your opinion, how suitable is the investigator-assessed swallowability method for young people aged 12–18 years?	In your opinion, how suitable is the investigator-assessed swallowability method for young people from 6–11 years?	In your opinion, rate the Visual Analogue Scale of 10 cm for the assessment of swallowability for young people	In your opinion, rate the 5-point Likert Scale without any icons for the assessment of swallowability for young people	In your opinion, rate the 5-point Likert Scale with other icons for the assessment of swallowability for young people	In your opinion, rate the YES–NO question for the assessment of swallowability for young people
7	2	10	10	10	10
8	10	10	10	10	8
8	8	7	8	8	8
2	6	8	6	2	8
3	6	9	8	7	3
6	10	8	3	8	6
6	7	9	9	9	8
5	8	4	6	5	2
8	10	6	6	10	5
7	9	9	7	6	7
8	9	8	7	7	5
6.18	7.82	8.00	7.27	7.45	6.36

The results indicate that the investigator-assessed swallowability method is suited for the younger age group from 6 to 11 years, while a Visual Analogue Scale of 10 cm is preferred for young people. 3.5. *Anticipated influence of cultural differences.*

The answers to the question on cultural differences are listed in Table 5.

**Table 5:** Cultural differences to be expected when young people from different countries will answer the questions in Tables 2, 3 and 4 and Fig. 2

Do you think that there are cultural differences to be expected when teenagers from different countries will answer the previous questions?
Yes, I expect big differences in teenagers
Yes, I expect big differences in teenagers
Yes, I expect big differences in teenagers
Yes, but I expect only minor differences in teenagers
Yes, but I expect only minor differences in teenagers
Yes, but I expect only minor differences in teenagers
Yes, but I expect only minor differences in teenagers
Yes, but I expect only minor differences in teenagers
Yes, but I expect only minor differences in teenagers
No, I expect no differences in teenagers

10 out of 11participants answered this question. 9 out of 10 participants expect differences, the majority expect minor differences when teenagers from different countries will answer the questions raised before, one participant expects no differences.

### Relevance of EMA-listed acceptability parameters

In the second part of the focus group meeting, the research group wanted to collect the opinion of the young people on the relevance of the 8 parameters of acceptability listed in the EMA guideline. This was done in two ways. First, the young people were asked to select the most and the least important parameter in their opinion. In a second step, they were asked to rank the 8 parameters. Also, these questions were asked a second time 5 weeks later.

**Fig. 3:**
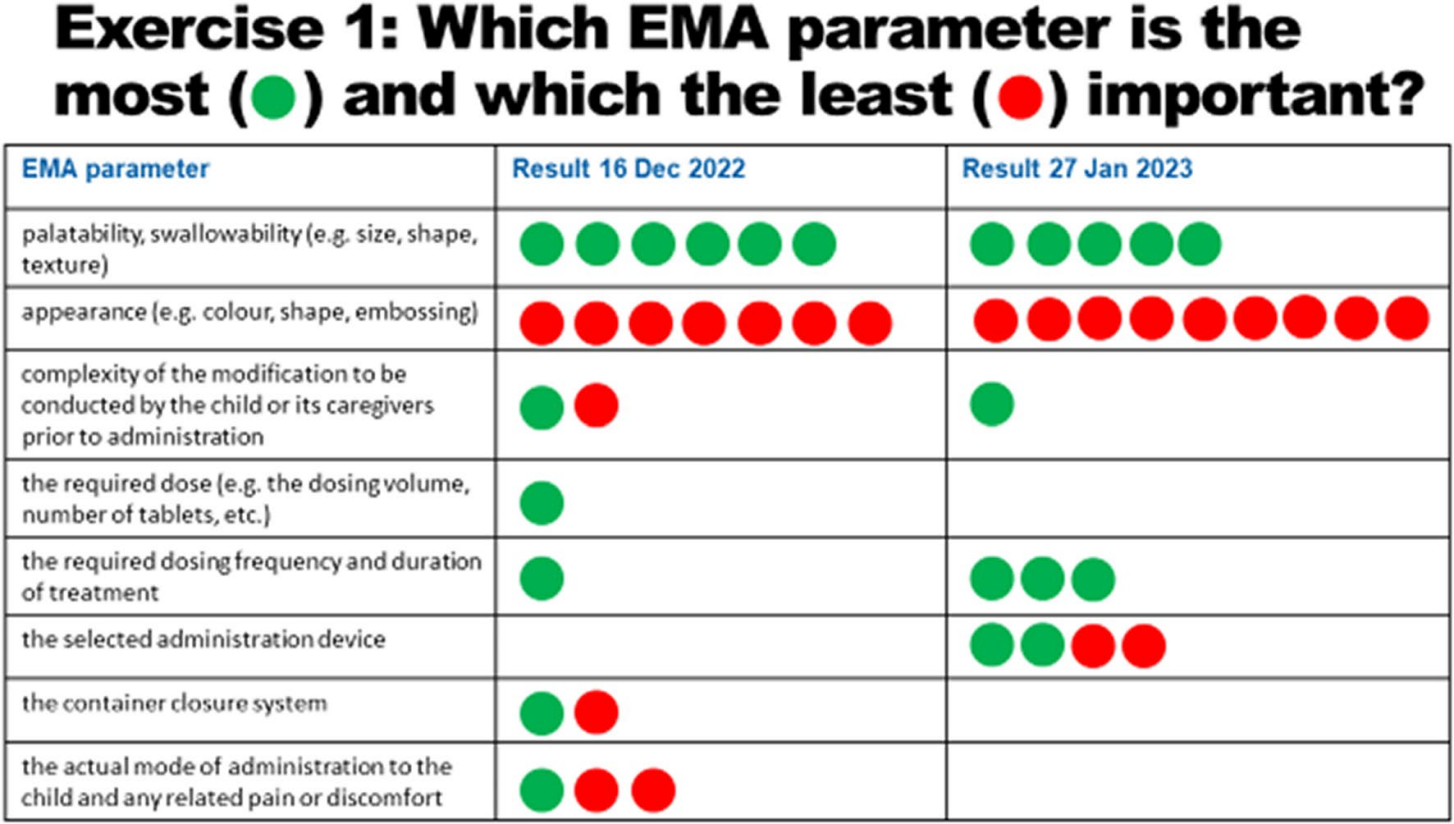
Selection of the most and least important EMA parameter

The answers to the question on which is the most important and which is the least important EMA-listed parameter for acceptability are shown in Fig. 3.

The results show that palatability and swallowability were considered to be the most relevant parameters when assessing acceptability and that appearance is the least important parameter defining acceptability. This outcome was the same when conducted in December 2022 and repeated in January 2023. But a little variance in the outcome can be detected: From December to January palatability and swallowability as most relevant aspects of acceptability lost one green point while the opinion on appearance as least relevant aspect increased by two red points.

**Fig. 4:**
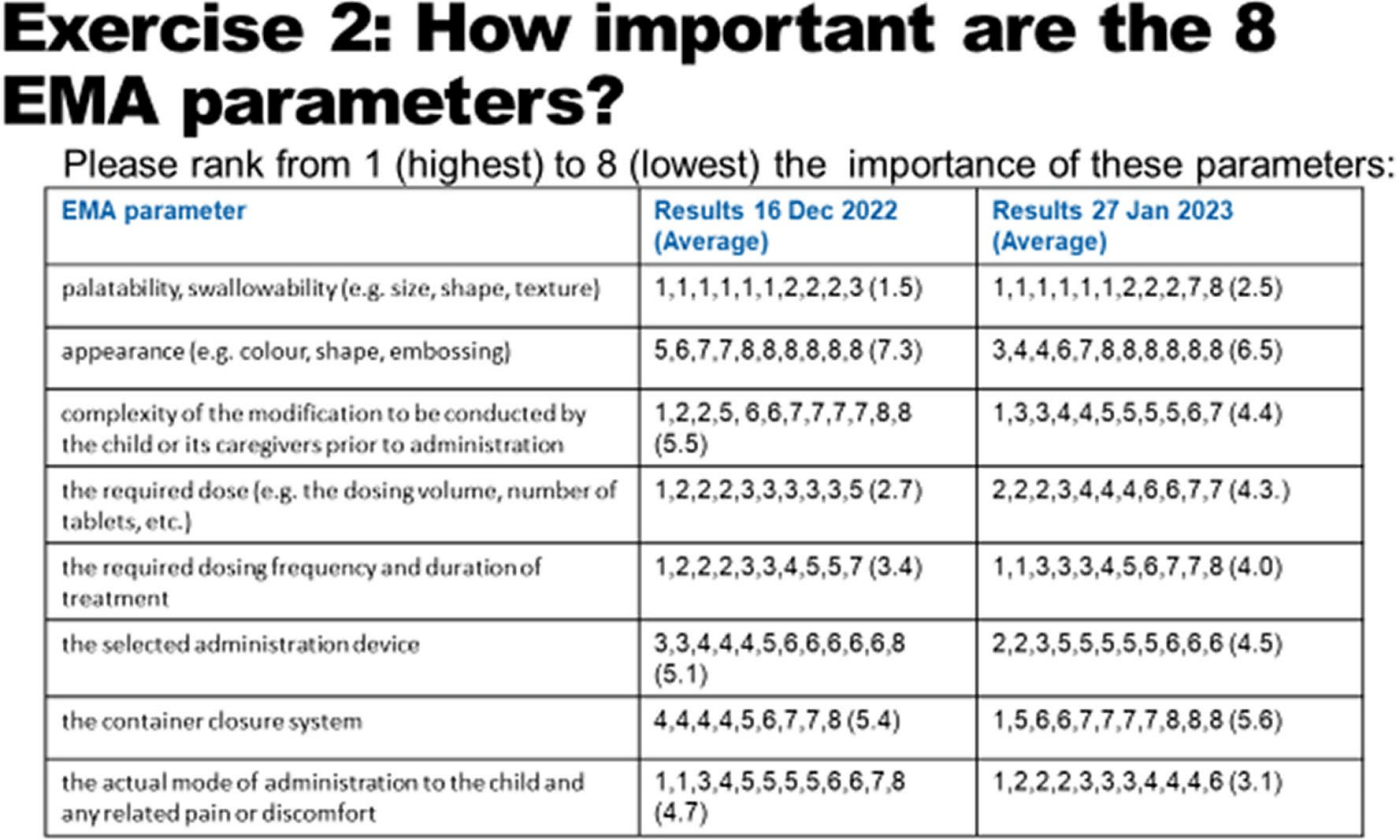
Ranking of the importance of all 8 EMA parameters

It must be noted that the method of opinion collection for both questions differed between these two occasions: In December during the joint focus group meeting the participants were asked to physically place colored dots/numbers behind the parameters, and in January 2023 they filled in an individual paper-based questionnaire at home. The results are given in Fig. 4.

The results show again that palatability and swallowability are the most important EMA parameters for the young people and appearance the least important parameter. As second choice “the actual mode of administration to the child and any related pain or discomfort” was chosen in both, the sticker method as well as the paper-based questionnaire method.

Beyond this clear choice, no significant differences in ranking between the parameters “Complexity of the modification to be conducted by the child or its caregivers prior to administration”, “The required dose (e.g., the dosing volume, number of tablets, etc.)”, “The required dosing frequency and duration of treatment”, and “The selected administration device” could be detected.

The relevance of the “Container closure system” was rated relatively low.

In general, the method of physical placing of coloured and numbered stickers showed weaknesses over the individual paper-based questionnaire, as not 11 ratings were given per parameter but sometimes more than 11 or less than 11 ratings. With the individual paper-based questionnaire 11 ratings per parameter were given, so it seems that this method was better suited to determine the ranking of the EMA acceptability parameters.

## Discussion

The benefit of involving patients as advisors in different aspects of the medicines development process is widely accepted [17]. Tailored methodology for the process of patient involvement is recommended considering the specifics of the project in which they will be part of (age, disease, topic of the patient involvement activity, etc.) [18–22]. But there is little experience and recommendation on how best to involve children and young people. The key objectives of this focus group-based investigation wereto gain experience in how best to involve young people—that are educated in the drug development process—as advisors to the acceptability assessment method in new paediatric dosage form development.

The used classification of children and adolescents in the questions asked (6–11 years and 12–18 years) corresponds to the common classification in paediatrics, according to ICH Guideline E11 [3].

To enable comprehensive information on the acceptability research methodology as well as direct observation of the reactions and responses to the questions, a face-to-face focus group meeting with a group of adolescents and young adults aged 16–23 years educated in the medicines development methodology was considered essential. The KIDS Barcelona YPAG was willing to collaborate with the research team on these topics because they felt that patient involvement in a research topic that is so relevant to develop a suitable paediatric medicine is important. National groups of young people of different age that are educated in clinical trials, clinical research and innovation like the KIDS Barcelona group are organized in the European YPAG network (eYPAGnet) [23]. This infrastructure enables the research community to gain access to the means and expertise of young people from different European YPAGs.

To ensure detailed planning and concrete understanding of the research approach, a “Young People Involvement Plan” was developed by the research group and the leader of the Spanish YPAG. This plan presented the background and rationale as well as a detailed description of the opinion gathering activities in the focus group. In an introductory presentation in lay language this “Young People Involvement Plan” was presented to the young people and triggered their interest in forming opinions on the different aspects raised. The concretely formulated questions on the composite endpoint on acceptability methodology were presented in an electronic questionnaire in English and in the adolescents and young adults’ Spanish mother tongue. It offered scales from 1 to 10 and defined age ranges to facilitate the expression of the patients’ ratings and recommendations. The handling of the electronic questionnaires was considered easy and was well accepted by all involved. Therefore, it seemed to be an appropriate and efficient method to get feedback on several different topics as the questions and answers could easily be tailored.

The method of selecting the most and least important aspects of acceptability by physically sticking colored dots behind the respective acceptability parameters presented on a flip chart at the wall worked well as all eleven red and all eleven green stickers were placed on the flip chart.

In the ranking exercise, however, the method of physical sticking of numbers behind the different parameters showed its limitations: Not always 11 ratings were given per parameter but sometimes more than 11 or less than 11 ratings, as the participants did not always remember where they had already placed their stickers. The most and least relevant and ranking exercises were repeated five weeks later with a paper-based questionnaire that the participants completed at home. Here the 11 participants delivered 11 complete rankings for the eight EMA parameters. Therefore, this method seemed to be better suited for a ranking exercise.

In the subsequent discussion among the young people during the focus group meeting it became clear that the ranking of the EMA parameters could be impacted by the participants’ disease and treatment experience.

The involvement of young patients in the medicines development and specifically in the acceptability method development was rated as very important by all young people. But opinions differed for example on the age as of which self-assessment method should start and which self-assessment method would be best for the respective age groups.

Interestingly, the young people rated swallowability and palatability as the most important acceptability elements in the EMA list of parameters while appearance—expected to be high-ranking as well—was the least important. The same results were found with both assessment methods.

This was the first investigation with a limited number of young people, from one country and with just one age group (16–23 years). Therefore, and based on the experiences made, the research team and other YPAGs members of eYPAGnet intent to replicate this patient involvement activity in further focus group meetings with more young people from different European countries and to include different age groups. As an alternative approach the research team considers developing a remote strategy with a video of the introductory method explanations and a toolkit to answer the questionnaires online. The results from the two different strategies will be compared. Once more data will be available from a larger number of young people from different regions and from different age groups, the research team will report on the experiences made with this method of young patient involvement and recommend the most efficient young patient contribution process in the acceptability assessment methodology. The composite endpoint on acceptability was validated based on a validation study in patients 1–6 months and 6–18 years. Publication of the results is currently ongoing.

## Conclusions

This young patient involvement project reinforced the need for systematically enabling the contributions of young people with knowledge about the medicines development process to the research and development processes. Such contribution can help to enable concrete patient-relevant input in the suitability of the acceptability assessment method developed by clinicians and researchers for new paediatric oral dosage forms aiming that the scales and methods are appropriate, suited, understandable, and well-designed for the relevant patient population.

### Supplementary Information

**Additional file 1.** GRIPP 2 Short form.

## Data Availability

All data generated or analysed supporting the conclusions of this article are included in this published article.

## Abbreviations

EMA: European Medicines Agency
eYPAGnet: European Young Person’s Advisory Groups Network
YPAG: Young Person’s Advisory Group
