# Supplementary material for: Young patients’ involvement in a composite endpoint method development on acceptability for paediatric oral dosage forms
Source: Res Involv Engagem. 2023 Nov 29;9:108. doi: 10.1186/s40900-023-00520-8 (PMC10688476; doi:10.1186/s40900-023-00520-8)
Supplement: Supplementary file 1 — Additional file 1. GRIPP 2 Short form. [file 40900_2023_520_MOESM1_ESM.pdf]

Additional File The GRIPP2 Short Form

Section and topic	Item
1: Aim<br><br>Report the aim of PPI in the study	An acceptability testing method combining the outcome of "swallowability" and "palatability" assessments to a "composite acceptability endpoint" was recently developed and assessed by 11 members of a Young Person's Advisory Group from Sant Joan de Déu Children's Hospital.
2: Methods<br><br>Provide a clear description of the methods used for patient and public involvement (PPI) in the study	PPI was performed during a focus group meeting with the Kids Barcelona (advisory group).<br>The importance of involving patients in the paediatric medicine development and in the acceptability method development was investigated with electronic questionnaires. Questions on how best to determine palatability and swallowability were asked. The relevance of all EMA-listed acceptability elements was assessed via coloured and numbered stickers and questionnaires.
3: Results<br><br>Outcomes—Report the results of PPI in the study, including both positive and negative outcomes	The results showed that the need for involvement of young people in the medicines and acceptability method development was rated high. The group worked out that a 5-point smiley Likert Scale is preferred for assessing acceptability by 6 – 11 year old patients, while a Visual Analogue Scale is preferred for collecting adolescents´ (12 – 18 years) opinion. The ranking of the EMA-listed acceptability elements showed that palatability and swallowability are the most relevant parameters.
4: Discussion and Conclusion<br><br>Outcomes—Comment on the extent to which PPI influenced the study overall. Describe positive and negative effects.	To ensure detailed planning and concrete understanding of the research approach, a "Young People Involvement Plan" was developed by the research group and the leader of the Spanish YPAG. This plan presented the background and rationale as well as a detailed description of the opinion gathering activities in the focus group. In an introductory presentation in lay language this "Young People Involvement Plan" was presented to the young people and triggered their interest in forming opinions on the different aspects raised.<br>The diversity of methodologies used in the project (focus group, questionnaires and ranking exercises) facilitated the opportunity to gather the assessment of the young people and to validate the results with a second assessment time point.
5: Reflections/ critical Perspective<br><br>Comment critically on the study, reflecting on the things that went well and those that did not, so others can learn from this experience	This work reinforced the need to involve young people in the medicines lifecycle, and specifically in this acceptability method development. This encouraged the authors to future expand the same methods to large number of YPAGs.
